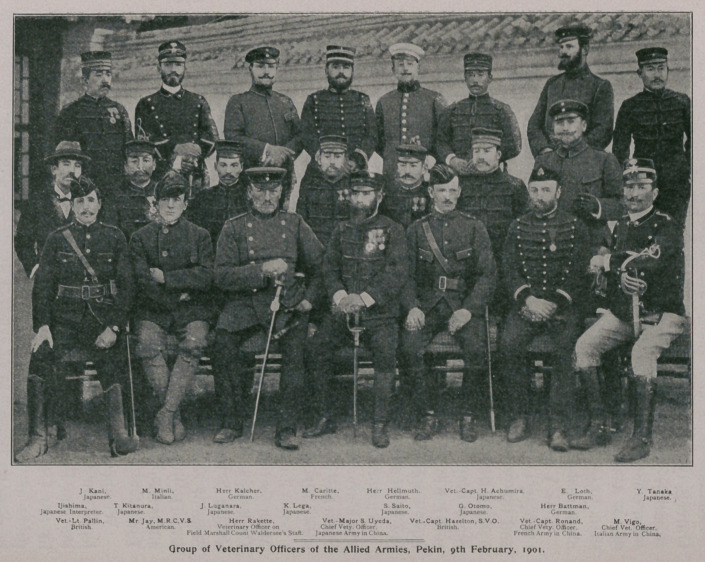# A National Disgrace

**Published:** 1901-08

**Authors:** 


### EDITORIALS.

### A NATIONAL DISGRACE.

We reproduce from the *Veterinary Record* of July 13th the photograph of a group of veterinary officers of the allied armies, with the key, which was taken at the Pekin International Club on February 9th.

It is a living picture of what we have been trying to represent to the Congress of the United States for some years, by printed facts, personal argument, and the indorsement of all the higher officers of practical experience in the United States Army.

This picture shows that the armies of Japan, Italy, Germany, France, and England (Russia is not represented in the picture) were accompanied by veterinary officers, uniformed officials, component parts of the military organization, whom we know to be empowered with all the obligations placed upon any officer, to be imbued with the esprit necessary for an officer to uphold his rank, and to be sure of a future recognition and the pension and the other rewards accorded to all officers of the army in which they serve.

In strong contrast the picture shows in one corner the American representative, a civilian; not an officer of the United States Army; not even an enlisted man; a simple hired civilian, who cannot receipt for the professional instruments which he uses, but must borrow them from the quartermaster under whom he serves; a civilian who is held responsible for his work, but can hold no one responsible for carrying out his instructions except through the personal interest and courtesy of the officer in command; a civilian who, if he performs his professional duty, is expected to endure the hardships, dangers, and discomforts, side by side with the soldier, but when his services terminate has no recognition, no retired pay, no pension for wounds, nor his wife and family any pension for death; and if he dies as Treacy, the veterinarian of the Eighth Cavalry, did, after twenty years' hard service in Indian wars, in a foreign country (in Cuba), is buried like a dog there, while a two-months' enlisted man's body is sent back to the United States at the expense of the Government.

We do not know Mr. Jay, M.R.C.V.S. M.R.C.V.S. (Member of the Royal College of Veterinary Surgeons) denotes that he is a reputable qualified veterinarian, or the Royal College of Great Britain would not have conferred his title upon him. We have not been able to learn that he is an American citizen. We know that he is not a member in good standing of any veterinary association in the United States. Why should an Englishman be sent as veterinarian of the United States Army to China?

Senators Hoar and Lodge are active supporters of Harvard University, which has one of the finest veterinary schools in America. Would not they rather see a born citizen of Massachusetts, educated at Harvard University, holding rank in the United States Army, an honor to their Commonwealth of Massachusetts, than to see the army's veterinary work done by a hired Englishman in slouch leggings and a fur cap?

New York State has the old New York-American Veterinary College, in New York City, which has graduated brilliant men, and the New York State Veterinary College, at Ithaca, subsidized by hundreds of thousands of dollars of the money from New York State taxpayers' pockets. Are not the people of New York State entitled to some consideration from Senators Platt and Depew, and representation in the United States Army, rather than an imported jay from a British Island?

Senators Quay and Penrose have a great veterinary school at the University of Pennsylvania; Senators Hanna's and Foraker's State has a reputable veterinary school at Columbus, Ohio; Senators Cullom's and Mason's constituents have two great veterinary schools in Illinois; Senators McMillan's and Burrows' State of Michigan has a veterinary school at Grand Rapids; and Senators Cockrell's and Vest's State of Missouri has a rising veterinary school at Kansas City which draws numbers of students from the State of Kansas, constituents of Senator Harris. Senators Allison and Dolliver represent a great agricultural State which supports an active school and educates excellent veterinarians at Ames, Iowa.

We appeal to the intelligence and sense of justice of our national legislators to give a proper recognition to the educated veterinarians in the United States and to accord them their proper rank in the United States Army. We appeal to the patriotism and pride of our legislators and ask them to place the veterinarian born and educated in the United States on an equal footing with the veterinarians of other countries. We appeal to the common sense of the American public to abandon the present obsolete method of employing civilian veterinarians in the army, and to establish an organized veterinary service in the army equal to or better than that of other countries.

## Figures and Tables

**Figure f1:**